# Mechanistic study of antisense long non-coding RNA *STEAP3-AS1* in breast cancer

**DOI:** 10.37349/etat.2026.1002380

**Published:** 2026-07-23

**Authors:** Jinquan Sun, Jiayong Cui, Tianlei Qiu, Guoshuang Shen

**Affiliations:** IRCCS Istituto Romagnolo per lo Studio dei Tumori (IRST) “Dino Amadori”, Italy; ^1^Graduate School of Qinghai University, Qinghai University, Xining 810016, Qinghai, China; ^2^Breast Disease Diagnosis and Treatment Center, Affiliated Hospital of Qinghai University, Xining 810000, Qinghai, China

**Keywords:** breast cancer, *STEAP3-AS1*, STEAP3, tumor suppressor, cuproptosis

## Abstract

**Aim:**

This study aims to investigate the expression and function of antisense long non-coding RNA (lncRNA) *STEAP3-AS1* in breast cancer (BC). Additionally, it explores STEAP3’s regulatory relationship with *STEAP3-AS1* and potential signaling pathways to provide a theoretical foundation for identifying novel therapeutic targets.

**Methods:**

Database prediction and collection of tissue samples were employed alongside a cell proliferation assay and Transwell migration and invasion assay to examine *STEAP3-AS1* expression levels in BC tissues and cell lines, as well as its impact on cellular functions. Statistical analyses were performed, including two-tailed Student’s *t*-test, Mann-Whitney *U* test and analysis of variance (ANOVA).

**Results:**

Both database and 9 paired clinical tissue sample results demonstrate that *STEAP3-AS1* expression was significantly downregulated in BC compared with normal breast tissues (*P* < 0.05). Compared with the estrogen receptor/progesterone receptor (ERPR)-negative (–)/human epidermal growth factor receptor-2 (Her2)-positive (+) subtype, the ERPR(+)/Her2(−) and ERPR(−)/Her2(−) subtypes exhibited a significantly lower expression level of *STEAP3-AS1* (*P* < 0.05). Overexpression of *STEAP3-AS1* markedly suppressed the proliferation, migration, and invasion capabilities of MDA-MB-231 and MCF-7 cells compared with negative controls (*P* < 0.05). Furthermore, *STEAP3-AS1* exhibited a positive synergistic effect with its sense strand STEAP3, inhibiting BC cell migration and invasion.

**Conclusions:**

This study is the first to demonstrate the tumor-suppressive role of *STEAP3-AS1* in BC. These findings provide novel insights into the mechanisms underlying BC progression and offer critical theoretical support for the development of new therapeutic strategies.

## Introduction

According to the 2022 GLOBOCAN cancer statistics, breast cancer (BC) ranked second in global incidence and fourth in mortality. Notably, female BC remains the leading cause of cancer-related mortality among women worldwide [1]. Based on molecular subtypes, BC is broadly classified into three categories: luminal, human epidermal growth factor receptor-2 (Her2)-positive, and triple-negative BC (TNBC). As per the 2024 Chinese Society of Clinical Oncology (CSCO) BC Guidelines, tumors expressing either estrogen receptor (ER) or progesterone receptor (PR) are classified as luminal type, whereas those negative (–) for ER, PR, and Her2 are defined as TNBC. Advances in clinical medicine and lifestyle interventions, combined with multimodal diagnostic and therapeutic strategies, have significantly improved clinical outcomes and quality of life for patients with BC [2, 3]. However, studies report suboptimal survival rates in metastatic cases [4, 5] and persistent adverse events following treatment [6]. Moreover, even early-stage diagnoses face challenges, including overtreatment-associated long-term complications [7] and significant survival disparities due to racial and intrinsic biological heterogeneity [8]. Some patients present with advanced-stage disease at initial diagnosis, while others experience recurrence despite timely intervention.

Delaying BC progression remains an urgent research priority. Current therapeutic approaches include surgery, neoadjuvant therapy, chemotherapy, and molecularly targeted therapies [9, 10]. Precision medicine [11] has introduced novel perspectives for personalized diagnosis and treatment, addressing interpatient heterogeneity and refining screening strategies for high-risk populations [12, 13]. Thus, elucidating the molecular mechanisms underlying BC pathogenesis is critical for identifying new therapeutic targets to mitigate disease progression.

Long non-coding RNAs (LncRNAs), a class of regulatory non-coding RNAs (alongside microRNAs, circular RNAs, and Piwi-interacting RNAs), are linear transcripts exceeding 200 nucleotides with limited protein-coding potential (open reading frames < 100 amino acids). Antisense lncRNAs, a subset of lncRNAs (which also include intronic, circular intronic, intergenic, sense, exonic circular, chimeric, chromatin-associated, and transcription start site-associated lncRNAs) [14], modulate gene expression via epigenetic, transcriptional, and post-transcriptional mechanisms, serving as tumor suppressors or oncogenic regulators [15]. Accumulating evidence implicates lncRNAs in tumor growth, migration, invasion, apoptosis, cell cycle regulation, drug resistance, and angiogenesis, with dysregulation reported in breast, ovarian, brain, liver, colorectal, and lung cancers, as well as leukemia, often correlating with poor prognosis [16–19].

Hypoxia-inducible factor (HIF) is a heterodimeric protein composed of oxygen-sensitive HIF-1/2/3α subunits and a constitutively expressed HIF-1β subunit. Under normoxia, HIF prolyl hydroxylases (PHDs) hydroxylate proline residues on the α subunit, enabling von Hippel-Lindau (VHL) protein-mediated ubiquitination and proteasomal degradation. During hypoxia, hydroxylation is suppressed, leading to non-hydroxylated α subunit accumulation, dimerization with HIF-1β, and binding to hypoxia-response elements (HREs) to activate transcription [20]. Factor inhibiting HIF-1 (FIH-1) further hydroxylates an asparagine residue in the HIF-α transactivation domain, blocking recruitment of coactivators p300 and CREB-binding protein (CBP) [21, 22]. HIF activation drives BC progression by regulating angiogenesis, metabolic reprogramming, cell motility, extracellular matrix remodeling, cancer stemness, therapy resistance, and immune evasion [23–26].

Hypoxic stress and/or HIF activation alter lncRNA expression in cancer cells, suggesting lncRNAs as key mediators of HIF-1α-regulated biological processes (BP). Conversely, certain hypoxia-responsive lncRNAs modulate HIF-1α activity [27, 28], indicating bidirectional crosstalk between HIF-1α and lncRNAs in hypoxia adaptation and tumor progression [29]. While aberrant HIF activation has been implicated in breast, gastric, pancreatic, renal, prostate, and colorectal cancers, the roles and mechanisms of HIF-associated lncRNAs in BC remain underexplored.

Through literature mining, The Cancer Genome Atlas (TCGA) database analysis, and preliminary experiments, our team identified *STEAP3-AS1*, a HIF-1α-associated antisense lncRNA with marked differential expression in BC tissues and cell lines. Prior studies revealed that *STEAP3-AS1* binds YTHDF2, an N6-methyladenosine (m6A) reader protein, to stabilize its sense-cognate STEAP3 mRNA, exerting a positive regulatory role in colorectal cancer [30].

This study systematically evaluates *STEAP3-AS1* expression patterns in BC tissues, malignant cell lines, and adjacent normal tissues, elucidating its functional impact on proliferation, migration, and invasion. We further investigate the regulatory interplay between *STEAP3-AS1* and STEAP3, employing rescue experiments to demonstrate that *STEAP3-AS1* suppresses malignant phenotypes by co-regulating STEAP3. Our findings delineate a novel lncRNA-mediated regulatory axis in BC progression, offering potential therapeutic targets.

## Materials and methods

### The Atlas of Non-coding RNA in Cancer (TANRIC) dataset

Comprehensive RNA-seq data for *STEAP3-AS1* across multiple cancers were obtained from TANRIC (https://www.tanric.org, version 2.2.2), an analytical platform built upon the TCGA database. This dataset incorporates samples from 20 cancer types in TCGA, 20 cancer lineages from Cancer Cell Line Encyclopedia (CCLE), and three additional independent datasets [31]. In total, it includes data from 837 BC tissue samples and 105 normal breast tissue samples.

### lnCAR database

The lnCAR database (https://lncar.renlab.org/) provides a comprehensive repository of human lncRNA expression profiles. This resource was constructed through systematic re-annotation of publicly available microarray data from over 57,000 samples. It enables users to investigate differentially expressed lncRNAs across diverse cancer types and to analyze the correlation between lncRNA expression levels and patient prognosis [32].

### Collection of tissue samples

BC tissue samples were obtained from 9 patients admitted to the Affiliated Hospital of Qinghai University (Qinghai Province, China) between April and September 2024. All enrolled patients were pathologically diagnosed with primary BC and underwent initial surgical resection without prior neoadjuvant radiotherapy. A total of 18 samples were collected, comprising both BC tissues and paired adjacent normal breast tissues taken 5 cm from the tumor margin, which served as controls. All specimens were snap-frozen in liquid nitrogen within 30 minutes after surgical resection and subsequently stored at –80°C for long-term preservation. Histological typing and quality control of the samples have been completed. This study received approval from the Ethics Committee of the Clinical Medical College of Qinghai University. Written informed consent was obtained from all participating individuals prior to their enrollment in the study.

Eligible participants were female patients aged 18–85 years with a histopathologically confirmed diagnosis of primary BC. All patients were treatment-naïve and scheduled to undergo initial curative surgical resection without prior neoadjuvant radiotherapy. Additionally, complete clinical records and a well-established postoperative follow-up data system were required for inclusion. Exclusion criteria comprised: (1) severe concurrent infectious diseases; (2) organic disorders of the gastrointestinal or immune systems; (3) metabolic syndrome; (4) communicable diseases; (5) decompensated dysfunction of major organs (e.g., heart, liver, or kidney); and (6) psychiatric conditions that could compromise compliance with treatment or study procedures.

### Cell culture

In this study, human BC cell lines (MDA-MB-231, SK-BR-3, MCF-7, and JIMT-1) and the non-tumorigenic human breast epithelial cell line MCF-10A were used (provided by Procell Life Science&Technology Co., Ltd). MDA-MB-231 represents TNBC, MCF-7 represents luminal-type Her2(−) BC, and SK-BR-3 and JIMT-1 represent Her2(+) ERPR(−) BC. The cell lines were authenticated by short tandem repeat (STR) profiling, which confirmed no evidence of human cell cross-contamination, and each cell line showed a 100% match with the corresponding cell line in the cell bank, indicating that the authentic cell lines were used. All cell lines were verified to be free from HIV-1, HBV, HCV, mycoplasma, bacteria, yeast, and fungal contamination.

Upon delivery, cryopreserved cells were promptly thawed and cultured in appropriate sterile media in suitable culture dishes. MDA-MB-231, SK-BR-3, MCF-7, and JIMT-1 cells were grown in high-glucose Dulbecco’s Modified Eagle Medium (DMEM) supplemented with 10% fetal bovine serum (FBS) and 1% penicillin-streptomycin (P/S). MCF-10A cells were cultured in DMEM/Ham’s F-12 Nutrient Mixture (DMEM/F12) supplemented with 5% horse serum (HS), 20 ng/mL epidermal growth factor (EGF), 0.5 μg/mL hydrocortisone, 10 μg/mL insulin, 1% non-essential amino acids (NEAA), and 1% P/S. All cell lines were maintained in a cell culture incubator at 37°C with 5% CO_2_ and 95% humidity.

### Plasmid and small interfering RNA (siRNA)

The *STEAP3-AS1* overexpression plasmid (pcDNA3.1-*STEAP3-AS1*) and the STEAP3 overexpression plasmid (pcDNA3.1-STEAP3) were designed and constructed by GenePharma Corporation. All plasmids contained EcoRI and BamHI restriction sites and an ampicillin resistance gene for prokaryotic selection. siRNAs targeting lncRNA *STEAP3-AS1* and STEAP3, along with corresponding overexpression plasmids, were synthesized by GenePharma Corporation (China). The siRNA sequences used for knockdown are listed below:

1. siRNA-*STEAP3-AS1*-1: 5'-GGCCGAUCAACACCUAAAU-3';
2. siRNA-*STEAP3-AS1*-2: 5'-GGAAGCCCUUAGAAUAAAU-3';
3. siRNA-STEAP3: 5'-GCUUCUAUGCCUACAACUU-3'.

### Quantitative reverse transcription polymerase chain reaction (qRT-PCR)

Total RNA was isolated from cultured cells and clinical tissue samples using the RNAsimple Total RNA Kit (TIANGEN, China). cDNA was synthesized immediately using the lnRcute lncRNA First-Strand cDNA Kit, followed by qPCR with the lnRcute lncRNA qPCR Kit (SYBR Green) (TIANGEN, China), according to the manufacturer’s protocols. The expression levels of *STEAP3-AS1* and STEAP3 were assessed, with β-tubulin serving as an internal reference gene. Relative gene expression was calculated using the 2^–ΔΔCT^ method after normalization to β-tubulin. All reactions were performed in triplicate. Primer sequences used are listed below:

1. *STEAP3-AS1*-F: 5'-GCTAGCTGCCTTTGACCTCC-3';
2. *STEAP3-AS1*-R: 5'-TAGGGAGCTGGTGAAGGTTTG-3';
3. STEAP3-F: 5'-CAGTCCTCACTGGGCTTTGT-3';
4. STEAP3-R: 5'-AAGGTGGGAGGCAGGTAGAA-3';
5. β-tubulin-F: 5'-CTCTGAAGCTGACCACACCA-3';
6. β-tubulin-R: 5'-GCCAGGCATAAAGAAATGGA-3'.

### Cell viability assay—MTS assay

Cell proliferation following modulation of *STEAP3-AS1* and STEAP3 expression was evaluated using the CellTiter 96^®^ AQueous One Solution Cell Proliferation Assay (MTS; Promega, USA). Briefly, cells were seeded in 96-well plates at a density of 5 × 10^3^ cells per well and cultured for 24, 48, and 72 hours. After each incubation period, the culture medium was aspirated, and 100 μL of a freshly prepared mixture containing 20 μL MTS reagent and 80 μL high-glucose DMEM (without supplements) was added to each well in the dark. The plates were then incubated at 37°C with 5% CO_2_ for 3 hours. Absorbance was measured at 490 nm using a multifunctional microplate reader (TECAN, Switzerland).

### Plate clonogenic assay

Cells were seeded in 6-well plates at a density of 5 × 10^2^ cells per well (ensuring absence of cell aggregates) in a total volume of 2 mL and incubated for 7–14 days. The culture medium was refreshed periodically with care to avoid disrupting cell growth. When the most individual clones contained ≥ 50 cells, the cells were gently washed with PBS and fixed with 4% paraformaldehyde for 30–60 minutes. After an additional PBS wash, the colonies were stained with 0.1% crystal violet for 20 minutes, followed by repeated PBS washes and air-drying. The plates were then imaged using a microscope, with both the entire plate and individual wells documented.

### Transwell migration and Matrigel invasion assays

For both assays, 5 × 10^4^ cells were resuspended in 200 μL of serum-free medium. In the migration assay, the cell suspension was directly seeded into the upper chamber of 24-well Transwell inserts (8 μm pore size; Corning, USA). For the invasion assay, Matrigel (Corning, USA) was diluted 1:7 with unsupplemented high-glucose DMEM, and 60 μL of the mixture was applied to the upper chamber of each insert, ensuring uniform coverage without air bubbles. The coated inserts were incubated at 37°C for 3 hours to allow gel polymerization, followed by 30 minutes of hydration. Cells were then seeded into the pre-coated inserts. The lower chamber of each well was filled with 500 μL of high-glucose DMEM containing 20% FBS as a chemoattractant. After 36 hours of incubation at 37°C in 5% CO_2_, non-migrated/non-invaded cells on the upper surface were carefully removed with a cotton swab. Cells that had migrated or invaded to the lower surface were fixed with 4% paraformaldehyde for 30 minutes, stained with 0.1% crystal violet for 30 minutes, and quantified under an optical microscope.

### Kyoto Encyclopedia of Genes and Genomes (KEGG) and Gene Ontology (GO) databases

The KEGG database (http://www.kegg.jp/) is a knowledge base for molecular-level characterization and analysis of biological systems, with pathway maps representing its core data resource. These maps systematically illustrate cellular and organismal functions through molecular interactions and reaction networks. The GO database (https://geneontology.org/) provides a standardized framework for functional annotation of genes and gene products, supporting enrichment analysis of molecular functions (MF), BP, and cellular components associated with target genes or gene sets [33, 34].

### Search Tool for Retrieval of Interacting Genes/Proteins (STRING) database

Protein-protein interaction analysis was conducted using the STRING database, version 12.0 (https://cn.string-db.org/). All interactions were retrieved with a medium confidence score threshold set at 0.40. The resulting interaction network was visualized using the default “show network as is” clustering option [35].

### Statistical analysis

Data are expressed as mean ± standard deviation (SD). Differences between two groups were evaluated using two-tailed Student’s *t*-test or Mann-Whitney *U* test. For multi-group comparisons, a one-way analysis of variance (ANOVA) was employed. Survival outcomes were assessed using univariate Cox regression analysis or the log-rank test, and survival curves were generated using the Kaplan-Meier (K-M) method. A two-tailed *P*-value of less than 0.05 was considered statistically significant. All statistical analyses were performed using GraphPad Prism (version 10.3.0; GraphPad Software, Inc., USA).

## Results

### Bioinformatics analysis of differential expression and survival in BC tissues

**Figure 1:**
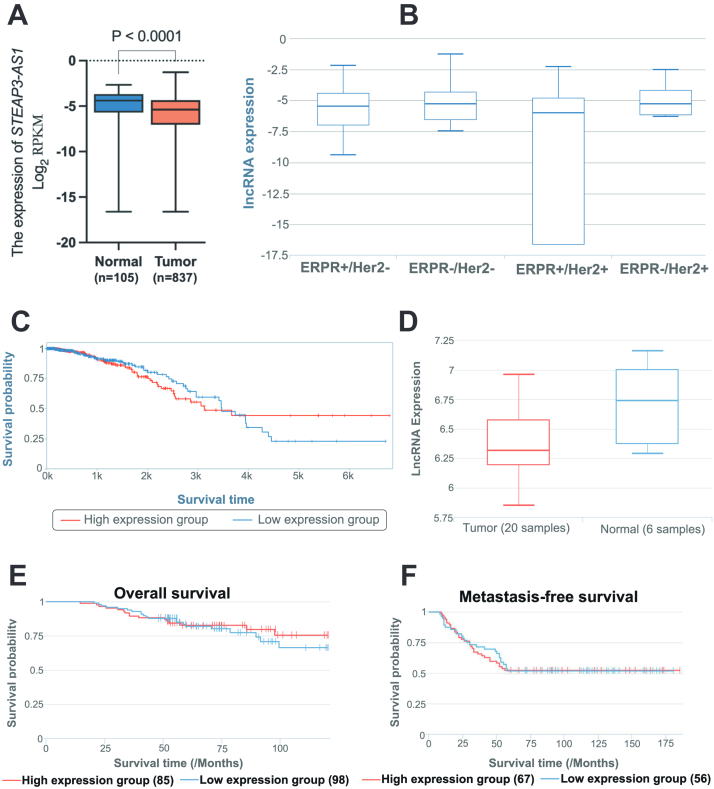
**Bioinformatics analysis of differential expression and survival in BC tissues.** (**A**) Analysis of the TCGA database revealed a comparative assessment of *STEAP3-AS1* expression levels in BC tissues versus adjacent normal breast tissues. *STEAP3-AS1* expression was significantly downregulated in BC tissues. (**B**) Evaluation using the TANRIC database comparing *STEAP3-AS1* expression levels across different molecular subtypes of BC. A significant reduction in *STEAP3-AS1* expression was observed in ERPR(+)/Her2(–) and ERPR(–)/Her2(–) subtypes relative to ERPR(–)/Her2(+) subtypes. (**C**) K-M survival analysis based on *STEAP3-AS1* expression levels in patients with BC was performed using the TANRIC database. The log-rank test indicated no statistically significant difference in overall survival between the high and low *STEAP3-AS1* expression groups among 837 patients. (**D**) Analysis via the LnCAR database compared *STEAP3-AS1* expression in TNBC tissues and adjacent normal tissues. *STEAP3-AS1* expression was significantly downregulated in TNBC tissues. (**E**, **F**) K-M survival analysis stratified by *STEAP3-AS1* expression levels in patients with BC was conducted using the LnCAR database. Cox proportional hazards model analysis showed no significant association between *STEAP3-AS1* expression groups and overall survival in 183 patients, and no significant association between *STEAP3-AS1* expression groups and MFS in 123 patients.

Using data from TCGA and the CCLE databases, we analyzed a total of 837 BC tissue samples and 105 normal breast tissue samples. The results demonstrated that *STEAP3-AS1* expression was significantly downregulated in BC tissues compared to normal breast tissues (*P* < 0.05) (Figure 1A). Furthermore, among different molecular subtypes of BC, the expression levels of *STEAP3-AS1* in the ERPR(+)/Her2(−) and ERPR(−)/Her2(−) subtypes were significantly lower compared with those in the ERPR(−)/Her2(+) subtype (*P* = 0.025854 < 0.05) (Figure 1B). To assess the prognostic significance of *STEAP3-AS1*, patients were stratified into high- and low-expression groups based on *STEAP3-AS1* levels, and K-M survival analysis was performed. Log-rank testing revealed no significant difference in overall survival (OS) between the high- and low-expression groups (*P* = 0.76079 > 0.05) (Figure 1C). Validation in an independent cohort (GSE33692, invasive ductal carcinoma) from the lncRNAs from Cancer Arrays (lnCAR) database confirmed that *STEAP3-AS1* expression was significantly reduced in tumor tissues compared to normal tissues (*P* = 0.0284 < 0.05) (Figure 1D). Additionally, Cox proportional hazards regression analysis of the cohort (GSE24450, BC) indicated that high *STEAP3-AS1* expression was associated with a negative Cox coefficient of –0.1589 < 0 and a hazard ratio (HR) of 0.8531 < 1, suggesting a potential protective effect of *STEAP3-AS1* in BC. However, no statistically significant differences were observed in OS (*P* = 0.6314 > 0.05) (Figure 1E). Cox proportional hazards regression analysis of the cohort (GSE37181, BC) indicated that high *STEAP3-AS1* expression was associated with a positive Cox coefficient of 0.0224 > 0 and an HR of 1.0226 > 1, suggesting a potential worse prognosis of *STEAP3-AS1* in BC. However, no statistically significant differences were observed in metastasis-free survival (MFS) (*P* = 0.9318 > 0.05) (Figure 1F).

### Expression profile of lncRNA *STEAP3-AS1* in BC tissues

#### Downregulation of *STEAP3-AS1* in BC tissues

**Figure 2:**
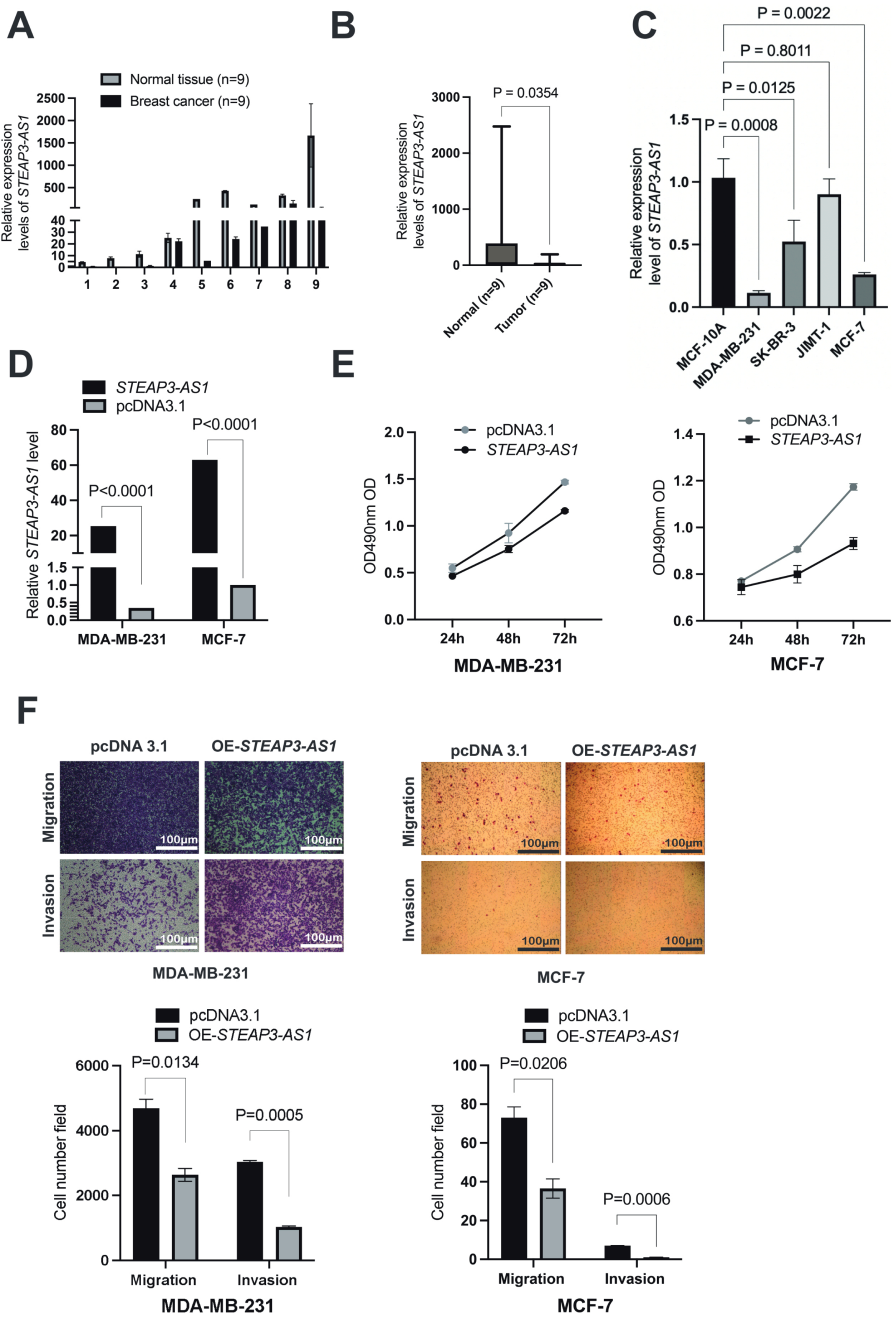
**Expression and functional analysis of *STEAP3-AS1* in BC cell lines.** (**A**) qRT-PCR analysis of *STEAP3-AS1* expression in nine paired human BC tissues and adjacent normal tissues. *STEAP3-AS1* expression was significantly downregulated in BC tissues compared with matched adjacent normal tissues in each pair. (**B**) qRT-PCR analysis of overall *STEAP3-AS1* expression across the nine paired human BC and adjacent normal tissue samples. *STEAP3-AS1* expression was significantly lower in BC tissues than in adjacent normal tissues across all samples. (**C**) Expression levels of *STEAP3-AS1* in different BC cell lines. *STEAP3-AS1* expression was significantly lower in MDA-MB-231 and MCF-7 cells. (**D**) Validation of *STEAP3-AS1* overexpression efficiency in BC cells. Transfection with pcDNA3.1-*STEAP3-AS1* resulted in an approximately several-fold increase in *STEAP3-AS1* expression in both MDA-MB-231 (73-fold) and MCF-7 (65-fold) cell lines. (**E**) Effect of *STEAP3-AS1* overexpression on BC cell proliferation. Overexpression of *STEAP3-AS1* significantly inhibited the proliferation of MDA-MB-231 and MCF-7 cells. (**F**) Effect of *STEAP3-AS1* overexpression on migration and invasion capabilities of BC cells. Overexpression of *STEAP3-AS1* significantly suppressed migration and invasion in both MDA-MB-231 and MCF-7 cells. Histological magnification: 10 × 10.

To investigate the differential expression of *STEAP3-AS1* between BC and normal mammary tissues, we collected paired fresh tissue samples from 9 patients undergoing surgical resection at Affiliated Hospital of Qinghai University between April and September 2024. Each pair consisted of primary invasive breast carcinoma and histologically normal breast tissue obtained ≥ 5 cm from the tumor margin. Total RNA was extracted from all specimens and reverse-transcribed into cDNA. qRT-PCR analysis revealed that the median expression level of *STEAP3-AS1* in normal breast tissues (6.49) was approximately 1.45-fold higher than that in matched tumor tissues (4.48). Statistical analysis confirmed a significant downregulation of *STEAP3-AS1* in malignant versus normal tissues (*P* < 0.05) (Figure 2A and B).

#### Clinicopathological characteristics of patients with BC

The clinicopathological parameters of all 9 enrolled patients with BC are summarized, including age, surgical classification, TNM stage, pathological grade, tumor diameter, Ki-67 index, and lymph node metastasis status (Table 1). The cohort comprised both TNBC and luminal-type BC cases, with TNBC accounting for 22% and luminal-type representing 78% of all cases.

**Table 1:** Clinical characteristics of patients with BC.

**Number**	**Age**	**Gender**	**Surgical classification**	**TNM staging**	**Pathological grade**	**Tumor diameter (cm)**	**Ki67 index (%)**	**Lymph node metastasis**
1	49	Female	Luminial B	T2N2M0	Medium differentiation	3.4	20	Yes
2	82	Female	TNBC	T2N0M0	High differentiation	2.2	5	No
3	44	Female	Luminial B	TisN0M0	/	3.7	5	No
4	49	Female	Luminial B	T2N0M0	Low differentiation	2.4	40	Yes
5	62	Female	Luminial B	T4N0M0	Medium differentiation	1.2	> 10	yes
6	49	Female	TNBC	T2N0M0	Low differentiation	2.3	40	Yes
7	55	Female	Luminial B	T4N1M0	Low differentiation	3.5	40	No
8	61	Female	Luminial B	T2N1M0	Medium differentiation	0.6	< 10	Yes
9	58	Female	Luminial A	T1N0M0	Medium differentiation	0.5	> 10	No

TNM: tumor, node, metastasis.

### Expression and functional analysis of lncRNA *STEAP3-AS1* in BC cell lines

#### Cell line selection based on *STEAP3-AS1* expression profiles

To establish appropriate cellular models for subsequent functional studies, we analyzed *STEAP3-AS1* expression patterns across multiple BC cell lines. Total RNA was extracted from human BC cell lines (MDA-MB-231, SK-BR-3, MCF-7, JIMT-1) and the normal mammary epithelial cell line (MCF-10A), followed by reverse transcription and qRT-PCR analysis. Comparative expression analysis revealed significant downregulation of *STEAP3-AS1* in malignant cell lines relative to MCF-10A controls. Relative to MCF-10A controls, SK-BR-3 cells exhibited a modest but statistically significant difference in expression (*P* < 0.05), whereas MDA-MB-231 and MCF-7 cells demonstrated more pronounced differential expression (*P* < 0.01). In contrast, JIMT-1 cells showed no significant difference (*P* = 0.8011) (Figure 2C). Given that the primary objective of this study was to investigate the functional consequences of *STEAP3-AS1* dysregulation, we prioritized cell lines exhibiting more substantial expression alterations—namely MDA-MB-231 and MCF-7—for subsequent loss-of-function and gain-of-function experiments.

#### Construction and validation of *STEAP3-AS1* overexpression plasmid in BC cells

To investigate the functional role of *STEAP3-AS1* in vitro, we designed and successfully constructed a *STEAP3-AS1* overexpression plasmid through GenePharma Corporation. The overexpression efficiency of the constructed vector was validated by transiently transfecting pcDNA3.1-*STEAP3-AS1* and pcDNA3.1 into MDA-MB-231 and MCF-7 cell lines. Twenty-four hours post-transfection, qRT-PCR analysis demonstrated that cells transfected with pcDNA3.1-*STEAP3-AS1* exhibited approximately 73-fold (MDA-MB-231) and 65-fold (MCF-7) higher *STEAP3-AS1* expression levels compared to the empty vector control group (*P* < 0.05) (Figure 2D). These results confirm the successful establishment of an efficient *STEAP3-AS1* overexpression system in both BC cell lines.

##### Overexpression of *STEAP3-AS1* inhibits proliferation in BC cell lines

To investigate the impact of *STEAP3-AS1* on cellular proliferation, we conducted MTS cell proliferation assays. MDA-MB-231 and MCF-7 cells were transiently transfected with either pcDNA3.1-*STEAP3-AS1* or pcDNA3.1. Twenty-four hours post-transfection, cells were seeded into 96-well plates for proliferation analysis. MTS reagent was added at 24, 48, and 72 hours after cell attachment, followed by 3 hours of incubation. Absorbance at 490 nm was measured using a microplate reader to generate growth curves. The results demonstrated that *STEAP3-AS1* overexpression significantly suppressed proliferation in both MDA-MB-231 and MCF-7 cell lines compared to vector controls (Figure 2E).

##### Overexpression of *STEAP3-AS1* suppresses migration and invasion in BC cell lines

To elucidate the relationship between *STEAP3-AS1* expression levels and metastatic potential in BC cells, we performed Transwell migration and Matrigel invasion assays using two BC cell lines. MDA-MB-231 and MCF-7 cells were transiently transfected with either pcDNA3.1-*STEAP3-AS1* or pcDNA3.1. Twenty-four hours post-transfection, cells were seeded into Transwell chambers and allowed to migrate/invade for 36 hours before termination of the assay. Quantitative analysis demonstrated that *STEAP3-AS1* overexpression significantly inhibited both migratory and invasive capacities in MDA-MB-231 and MCF-7 cells compared to vector controls (*P* < 0.05) (Figure 2F). These findings suggest a potent suppressive role of *STEAP3-AS1* in BC metastasis.

#### Validation of *STEAP3-AS1* knockdown efficiency in BC cells

**Figure 3:**
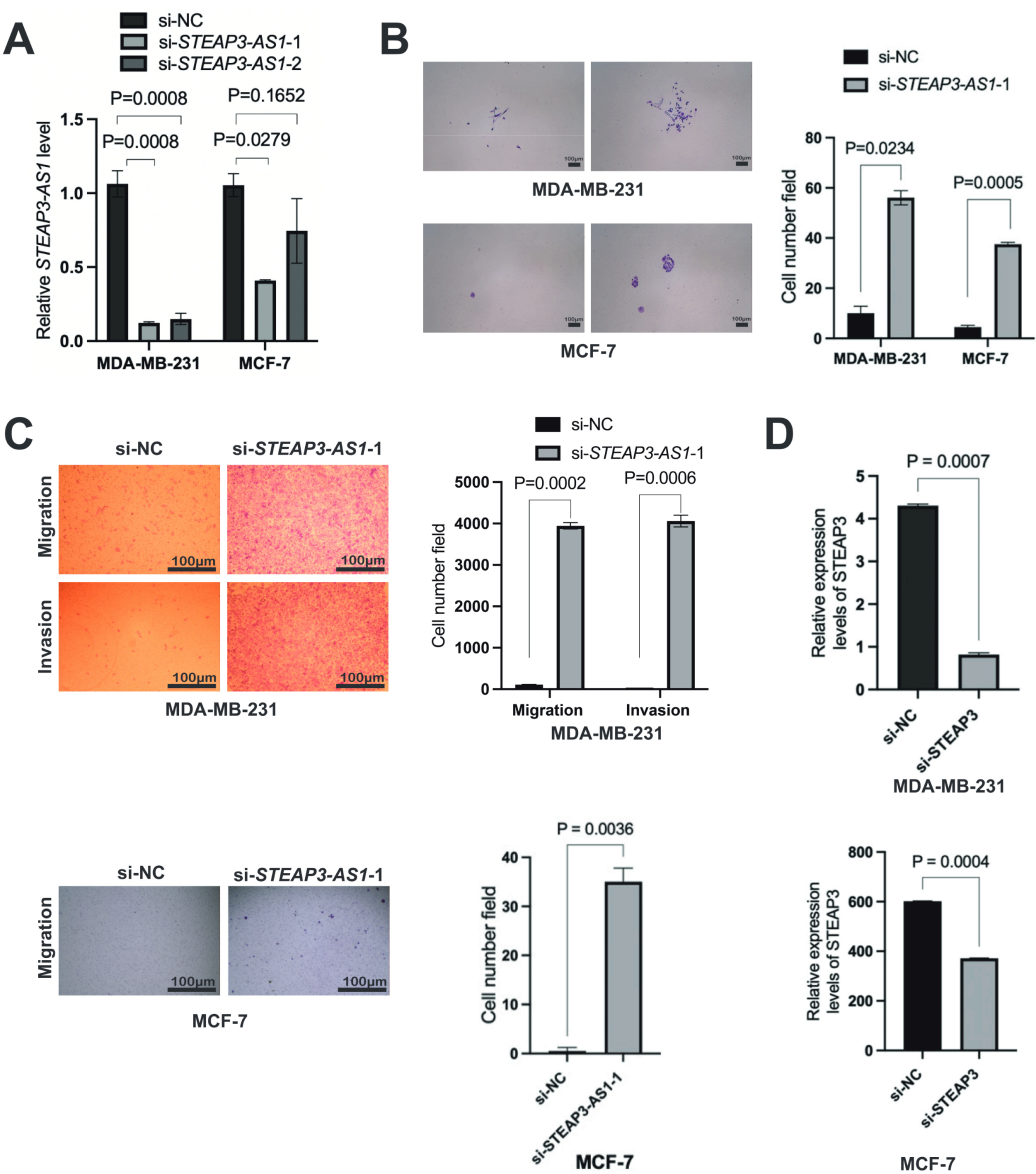
**Expression and functional analysis of *STEAP3-AS1* in BC cell lines.** (**A**) Evaluation of *STEAP3-AS1* knockdown efficiency in BC cells. Transfection with si-*STEAP3-AS1*-1 significantly reduced *STEAP3-AS1* expression in both MDA-MB-231 and MCF-7 cell lines. (**B**) Effect of *STEAP3-AS1* knockdown on BC cell proliferation. Knockdown of *STEAP3-AS1* significantly promoted the proliferation of MDA-MB-231 and MCF-7 cells. Magnification: 10 × 4. (**C**) Effect of *STEAP3-AS1* knockdown on migration and invasion capabilities of BC cells. Knockdown of *STEAP3-AS1* significantly enhanced migration and invasion in MDA-MB-231 and MCF-7 cells. Histological magnification: 10 × 10. (**D**) Validation of STEAP3 knockdown efficiency in BC cells. Transfection with si-STEAP3 significantly decreased STEAP3 expression in MDA-MB-231 and MCF-7 cell lines.

To investigate the functional consequences of *STEAP3-AS1* depletion, we designed and synthesized two siRNAs (si-*STEAP3-AS1*-1 and si-*STEAP3-AS1*-2) through GenePharma Corporation. MDA-MB-231 and MCF-7 cells were transiently transfected with either si-*STEAP3-AS1*-1, si-*STEAP3-AS1*-2, or negative control siRNA (si-NC). Following 24-hour incubation, total RNA was extracted for reverse transcription and subsequent qRT-PCR analysis to evaluate knockdown efficiency. The results demonstrated significant *STEAP3-AS1* suppression in MDA-MB-231 cells transfected with both si-*STEAP3-AS1*-1 (88% knockdown efficiency, *P* < 0.05) and si-*STEAP3-AS1*-2 (86% knockdown efficiency, *P* < 0.05) compared to si-NC. In MCF-7 cells, only si-*STEAP3-AS1*-1 achieved statistically significant knockdown (59% efficiency, *P* < 0.05), while si-*STEAP3-AS1*-2 showed no significant effect (*P* > 0.05). Based on its superior knockdown performance across both cell lines, si-*STEAP3-AS1*-1 was selected for subsequent functional studies (Figure 3A).

##### Knockdown of *STEAP3-AS1* promotes proliferation in BC cell lines

To further validate the functional impact of *STEAP3-AS1* depletion on cellular proliferation, we performed colony formation assays. MDA-MB-231 and MCF-7 cells were transiently transfected with si-*STEAP3-AS1*-1 or si-NC. Twenty-four hours post-transfection, cells were seeded into 6-well plates and allowed to form colonies for 14 days, followed by fixation and crystal violet staining. Quantitative analysis revealed that *STEAP3-AS1* knockdown significantly enhanced proliferative capacity in both cell lines, as evidenced by increased cell numbers and higher colony formation rates compared to si-NC (*P* < 0.05) (Figure 3B). These results demonstrate that *STEAP3-AS1* silencing promotes BC cell proliferation, consistent with its putative tumor-suppressive role.

##### Knockdown of *STEAP3-AS1* enhances migration and invasion in BC cell lines

To further investigate the functional relationship between *STEAP3-AS1* depletion and metastatic potential, we conducted Transwell migration and Matrigel invasion assays in BC cell lines. MDA-MB-231 and MCF-7 cells were transiently transfected with either si-*STEAP3-AS1*-1 or si-NC. Twenty-four hours post-transfection, cells were seeded into Transwell chambers and allowed to migrate/invade for 36 hours before assay termination. Quantitative analysis demonstrated that *STEAP3-AS1* knockdown significantly increased both migratory and invasive capacities, as evidenced by elevated numbers of cells traversing the Transwell membrane compared to si-NC (*P* < 0.05) (Figure 3C). These findings provide compelling evidence that *STEAP3-AS1* silencing promotes metastatic behavior in BC cells.

#### LncRNA *STEAP3-AS1* positively correlates with STEAP3 to suppress BC cell functions

##### Alterations in STEAP3 expression modulate BC cell migration and invasion

Previous studies have demonstrated that lncRNA *STEAP3-AS1* directly binds YTHDF2, an m6A-modified RNA reader protein, to prevent degradation of its cognate sense strand STEAP3 mRNA, thereby exerting a positive regulatory effect on STEAP3 in colorectal cancer. We therefore sought to investigate whether this regulatory mechanism is conserved in BC cells.

To examine the functional consequences of STEAP3 expression modulation, we first manipulated STEAP3 mRNA levels through both overexpression and knockdown approaches. An STEAP3 overexpression plasmid and si-STEAP3 were designed and constructed by GenePharma Corporation. Knockdown efficiency was validated by transiently transfecting si-STEAP3 or si-NC into MDA-MB-231 and MCF-7 cell lines, followed by qRT-PCR analysis 24 hours post-transfection. The results demonstrated significant downregulation of STEAP3 expression in both MDA-MB-231 (81% efficiency, *P* < 0.05), and MCF-7 (38% efficiency, *P* < 0.05) cells transfected with si-STEAP3 compared to si-NC (Figure 3D), confirming the efficacy of our knockdown system. These findings establish a robust experimental platform for subsequent functional investigations of STEAP3 in BC progression.

**Figure 4:**
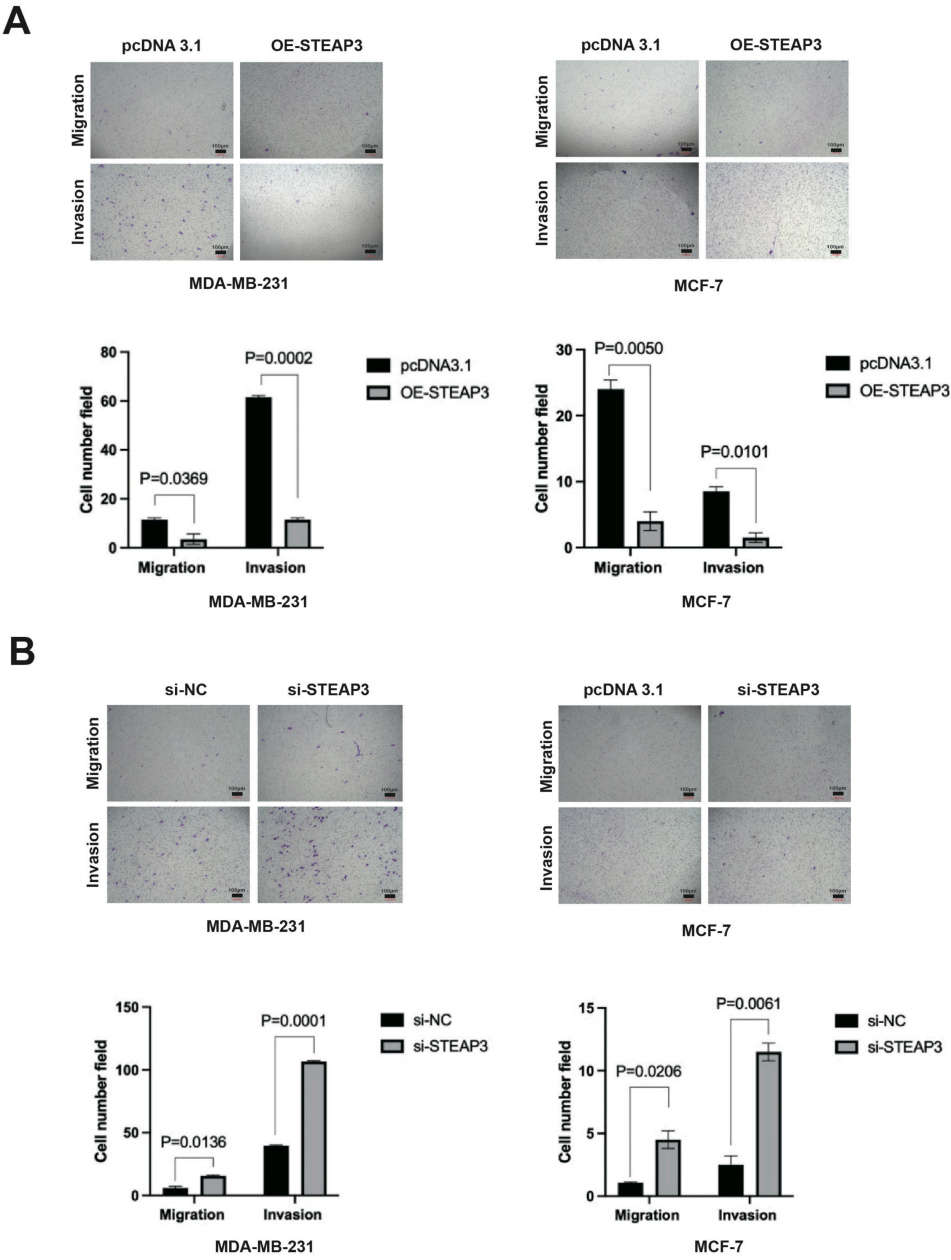
**Effects of STEAP3 overexpression and knockdown on migration and invasion capabilities of BC cells.** (**A**) Overexpression of STEAP3 significantly suppressed migration and invasion in MDA-MB-231 and MCF-7 cells. *P* < 0.05. (**B**) Knockdown of STEAP3 significantly promoted migration and invasion in both MDA-MB-231 and MCF-7 cells. *P* < 0.05. Magnification: 10 × 4.

To further elucidate the functional relationship between STEAP3 expression dynamics and metastatic potential, we conducted parallel Transwell migration and Matrigel invasion assays in both BC cell lines. MDA-MB-231 and MCF-7 cells were transiently transfected with either si-STEAP3, si-NC, pcDNA3.1-STEAP3, or pcDNA3.1. Following 24-hour incubation, transfected cells were seeded into Transwell chambers and allowed to migrate/invade for 36 hours before assay termination. Quantitative analysis revealed two key findings: First, STEAP3 knockdown significantly enhanced metastatic potential, with both cell lines exhibiting increased numbers of migrated and invaded cells through the Transwell membrane compared to si-NC (*P* < 0.05). Second, STEAP3 overexpression produced the opposite effect, markedly reducing migratory and invasive cell numbers relative to empty vector-transfected controls (*P* < 0.05) (Figure 4A and B).

##### *STEAP3-AS1* positively correlates with STEAP3 expression in BC cells

**Figure 5:**
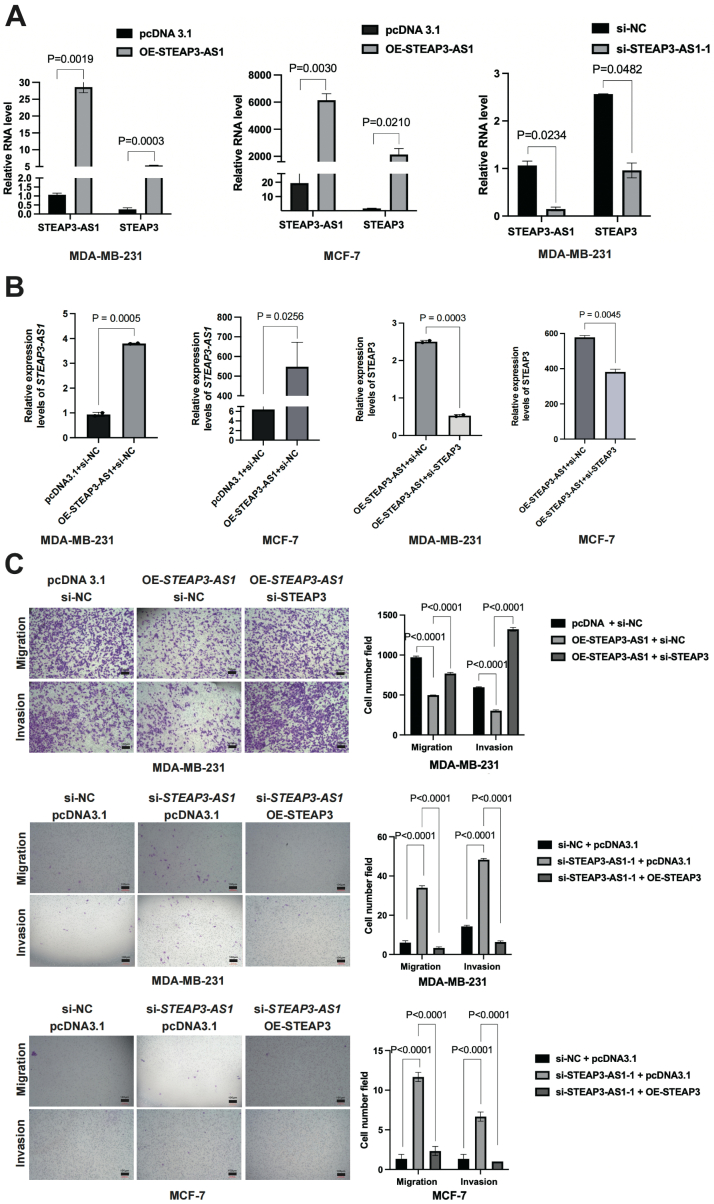
***STEAP3-AS1* positively regulates STEAP3 mRNA expression in BC cells.** (**A**) Relative expression levels of *STEAP3-AS1* and STEAP3 in control, *STEAP3-AS1*-overexpressing, and *STEAP3-AS1*-knockdown groups were detected by qPCR in BC cells. In both MDA-MB-231 and MCF-7 cells, *STEAP3-AS1* expression was significantly increased in the overexpression group, which led to a significant upregulation of STEAP3 expression. In MDA-MB-231 cells, *STEAP3-AS1* expression was significantly decreased in the knockdown group, resulting in a marked downregulation of STEAP3 expression. (**B**) Overexpression of *STEAP3-AS1* combined with knockdown of STEAP3 downregulated STEAP3 mRNA expression. (**C**) Overexpression of *STEAP3-AS1* together with knockdown of STEAP3 partially reversed the inhibitory effect of *STEAP3-AS1* on migration and invasion of BC cells. Conversely, knockdown of *STEAP3-AS1* combined with overexpression of STEAP3 partially attenuated the promotive effect of *STEAP3-AS1* knockdown on cell migration and invasion. Magnification: 10 × 4.

To investigate the association between *STEAP3-AS1* and STEAP3 in BC cells, we transfected MDA-MB-231 and MCF-7 cells with either pcDNA3.1-*STEAP3-AS1* or pcDNA3.1; and siRNA-*STEAP3-AS1*-1 or si-NC. Twenty-four hours post-transfection, total RNA was extracted for reverse transcription and subsequent qRT-PCR analysis of *STEAP3-AS1* and STEAP3 expression levels. The results demonstrated a consistent positive correlation: *STEAP3-AS1* overexpression significantly increased both *STEAP3-AS1* and STEAP3 mRNA levels compared to empty vector controls (*P* < 0.05); and *STEAP3-AS1* knockdown significantly decreased expression of both transcripts relative to si-NC controls (*P* < 0.05) (Figure 5A). These findings confirm that *STEAP3-AS1* positively correlates with STEAP3 in BC cells, consistent with previous reports in colorectal cancer [30].

#### Rescue experiments demonstrate STEAP3 potentially mediates *STEAP3-AS1*’s regulatory effects on BC cell migration and invasion

To further validate the positive correlation between *STEAP3-AS1* and STEAP3, we performed reciprocal rescue experiments in both BC cell lines. First, cells were co-transfected with pcDNA3.1-*STEAP3-AS1* and siRNA-STEAP3. This combined treatment resulted in significant downregulation of STEAP3 mRNA expression (*P* < 0.05) (Figure 5B), and partial attenuation of *STEAP3-AS1* overexpression-induced suppression of cell migration and invasion. Conversely, co-transfection with siRNA-*STEAP3-AS1*-1 and pcDNA3.1-STEAP3 led to successful STEAP3 upregulation, and partial reversal of *STEAP3-AS1* knockdown-induced enhancement of metastatic potential (*P* < 0.05) (Figure 5C). Collectively, these rescue experiments suggest that in both MDA-MB-231 and MCF-7 BC cells, lncRNA *STEAP3-AS1* likely modulates cellular migration and invasion primarily through its positive correlation with STEAP3 expression.

#### Bioinformatics analysis of downstream targets and potential pathways

**Figure 6:**
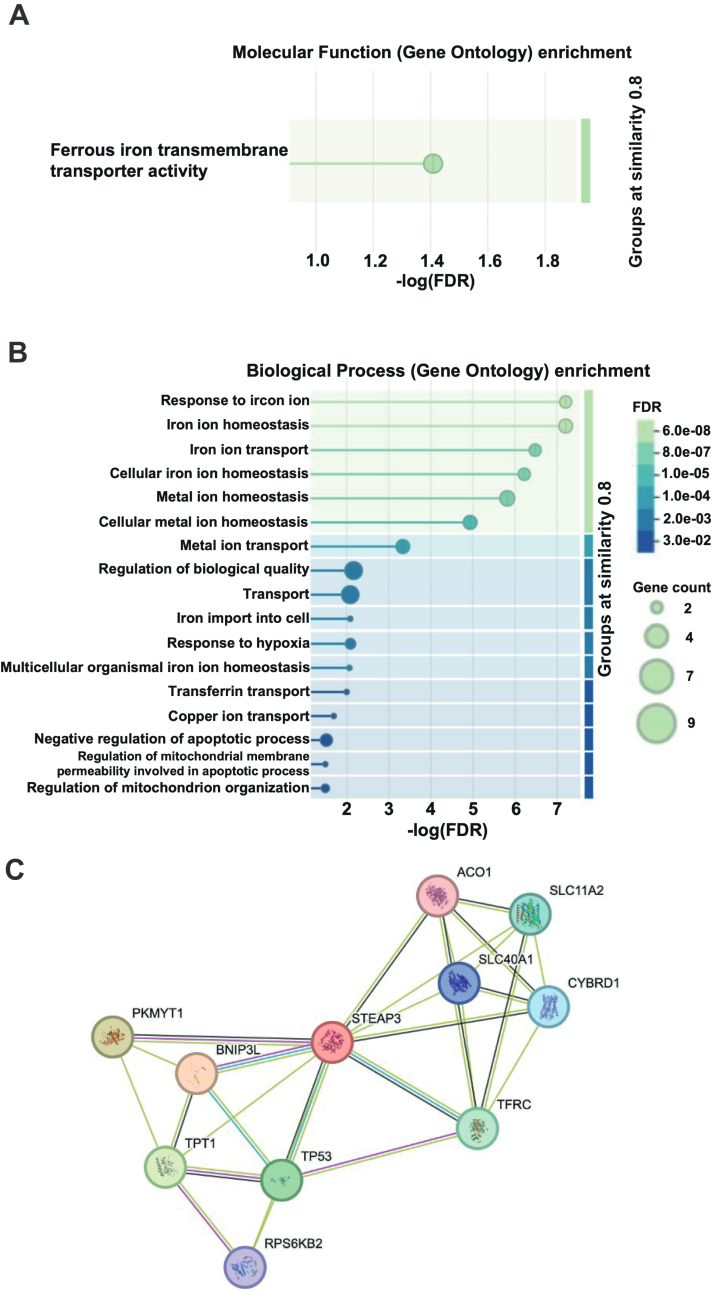
**Enrichment analysis of human STEAP3 using the GO database and protein-protein interaction network prediction for human STEAP3 using the STRING database.** (**A**) Molecular functions (MF). (**B**) Biological processes (BP). Terms with a false discovery rate (FDR) < 0.05 were considered statistically significant. (**C**) A protein-protein interaction network for STEAP3 was predicted using the STRING database.

To elucidate the molecular mechanisms underlying *STEAP3-AS1* and STEAP3-mediated regulation of BC cell biology, we conducted comprehensive bioinformatics analyses. Using the GO database, we predicted the MF and BP of human STEAP3. The results demonstrated that STEAP3, functioning as a metalloreductase, facilitates Fe^3+^ and Cu^2+^ reduction, participates in iron uptake by transferrin in erythrocytes, and maintains copper homeostasis. Additionally, STEAP3 appears to be involved in apoptosis regulation and mitochondrial function (Figure 6A and B). The KEGG pathway analysis revealed that STEAP3 may be associated not only with the ferroptosis pathway [36, 37] but also potentially functions downstream of the p53/TP53 signaling pathway to promote exosome secretion [38–44] (Ferroptosis—Reference pathway (map04216) [Internet]. Kyoto: KEGG; c2026 [cited 2026 Apr 22]. Available from: https://www.kegg.jp/pathway/map04216) (p53 signaling pathway—Reference pathway (map04115) [Internet]. Kyoto: KEGG; c2026 [cited 2026 Apr 22]. Available from: https://www.kegg.jp/pathway/map04115). Furthermore, protein-protein interaction network prediction via the STRING database identified the top five STEAP3-interacting proteins: (1) BNIP3L, an apoptosis-related protein involved in mitochondrial protein catabolism; (2) PKMYT1, a specific CDC2 inhibitory kinase that negatively regulates G2-M phase transition through CDK1 phosphorylation; (3) TPT1, a tumor-associated protein participating in calcium binding and microtubule stabilization; (4) TP53, a tumor suppressor inducing growth arrest or apoptosis; and (5) TFRC, a transferrin receptor protein (Figure 6C). Taken together, these bioinformatics findings support the hypothesis that *STEAP3-AS1*, through its positive correlation with STEAP3, may influence BC cell proliferation, migration, invasion, and apoptosis via multiple mechanisms including: (1) ferroptosis and cuproptosis pathways, (2) interaction with CDK1 and related proteins, and (3) promotion of exosome secretion downstream of the p53 signaling pathway.

## Discussion

Antisense lncRNAs, a distinct subclass of lncRNAs, have been increasingly recognized for their critical regulatory roles in BC progression, either through upregulation or downregulation [45]. For instance, Jiang et al. [46] demonstrated that the DNA damage-induced antisense lncRNA *DDIT4-AS1* is highly expressed in TNBC cells, where it stabilizes DDIT4 mRNA to induce autophagy, thereby promoting TNBC progression and chemoresistance. Similarly, Niknafs et al. [47] reported that the ER-regulated antisense lncRNA *DSCAM-AS1* is overexpressed in ER(+) tumors, where it interacts with heterogeneous nuclear ribonucleoprotein-L (hnRNP-L) to drive BC progression and tamoxifen resistance. Min et al. [48] identified *HIF1A-AS2* as an upregulated lncRNA in TNBC cells, where it modulates mitochondrial ribosomal small subunit protein (MRPS23) expression, influencing cellular processes and paclitaxel sensitivity. Liu et al. [49] found that the hyaluronan-mediated motility receptor (HMMR)-antisense lncRNA *HMMR-AS1* is elevated in basal-like BC (BLBC), where it regulates HMMR to promote BLBC progression. Additionally, Sun et al. [50] observed that *MEG3* is downregulated in BC samples; its overexpression suppresses malignant behavior by reducing MDM2 transcription, thereby stabilizing and activating p53.

Numerous antisense lncRNAs have been implicated in HIF-related pathways in malignancies. Zhou et al. [30] demonstrated that hypoxia-induced HIF-1α transcriptionally upregulates lncRNA *STEAP3-AS1* in colorectal cancer cells, where it prevents STEAP3 mRNA degradation, activating the Wnt/β-catenin pathway to drive tumor progression. Zheng et al. [51] reported that the HIF-1α antisense lncRNA *HIFAL* is upregulated in BC tissues, forming a positive feedback loop with HIF-1α to enhance its transcriptional activity and promote glycolysis under hypoxic conditions. Based on literature reviews, TCGA database analyses, and preliminary experiments, our research group identified *STEAP3-AS1* as a HIF-1α-associated antisense lncRNA with well-documented experimental support, exhibiting significant differential expression in BC tissues and cell lines. Our findings reveal that *STEAP3-AS1* is markedly downregulated in BC, particularly in luminal and TNBC subtypes. Functional assays demonstrate that *STEAP3-AS1* overexpression suppresses proliferation, migration, and invasion, whereas its knockdown enhances these malignant phenotypes.

Antisense lncRNAs are transcribed from the opposite strand of protein-coding genes [52]. They regulate gene expression through diverse mechanisms: (1) interacting with DNA, RNA, or proteins through recruiting/removing chromatin-modifying factors, or through histone modifications and DNA methylation inducing epigenetic changes [53]; (2) modulating transcription [54]; (3) post-transcriptionally influencing RNA splicing, mRNA stability, subcellular localization, or translation [55]; (4) acting as positive/negative regulators of protein-coding genes [56]; (5) and as a regulator of gene expression [50]. Regarding their complementary sense-strand mRNAs, antisense lncRNAs can exert control via: (1) RNA-DNA duplex/triplex formation (transcriptional regulation) [57]; (2) RNA-RNA base pairing (post-transcriptional regulation) [58, 59]; or (3) molecular decoy mechanisms, where they sequester or recruit proteins to modulate gene expression at multiple levels [60, 61].

In our study, *STEAP3-AS1* exhibited a positive regulatory effect on its sense-strand STEAP3 mRNA in MDA-MB-231 and MCF-7 BC cells, consistent with prior reports [30]. Subsequent functional assays revealed that STEAP3 suppresses migration and invasion. Rescue experiments confirmed that *STEAP3-AS1* upregulation-mediated tumor suppression was reversed by STEAP3 knockdown, and *STEAP3-AS1* knockdown-induced oncogenic effects were attenuated by STEAP3 overexpression. These results establish *STEAP3-AS1* as a tumor suppressor that positively cooperates with STEAP3 to inhibit BC cell proliferation, migration, and invasion.

Study limitations: (1) First, the sample size (*n* = 9 pairs) is relatively modest, which may limit the statistical power to detect small effect sizes and make it difficult to capture the expression characteristics across different pathological types and stages of BC. Second, the cohort was derived from a single institution, potentially introducing selection bias. Third, the findings were not validated in an independent external cohort. Thus, the applicability of this conclusion is limited. Nevertheless, the consistency of our results with TCGA data (*n* = 942) and InCAR data (*n* = 26) strengthens the robustness of the conclusions. Future studies with larger, multicenter cohorts are warranted to validate these findings. (2) “adjacent normal breast tissues” were defined as samples collected 5 cm from the tumor margin without histopathological confirmation, raising the possibility of microscopic tumor infiltration or premalignant lesions. This may have led to an underestimation of the true *STEAP3-AS1* expression differences. Nevertheless, the 5 cm distance is widely accepted in BC studies as a safe margin for histologically normal tissue [62–65]. Importantly, the observed expression differences remained significant (*P* < 0.05), and any potential contamination would only attenuate the effect size, suggesting that our findings are likely conservative. (3) The publicly available databases employed in this study did not provide sufficient data to investigate the association between *STEAP3-AS1* expression and clinical indicators such as disease-free survival (DFS) and treatment response. Accordingly, our analysis was limited to OS and MFS using the InCAR database (Figure 1E and F). The insufficient assessment of survival endpoints represents a limitation of the current study, which will need to be addressed in future research by collecting larger samples and long-term follow-up data. (4) We acknowledge that the regulatory relationship between *STEAP3-AS1* and STEAP3 was validated only at the mRNA level, and the corresponding protein-level changes were not confirmed by Western blotting (WB). Although mRNA expression often correlates with protein abundance, post-transcriptional regulation may lead to discrepancies between transcript and protein levels. Therefore, whether the observed upregulation of STEAP3 mRNA by *STEAP3-AS1* translates into increased STEAP3 protein expression remains to be determined. Future studies employing WB analysis are warranted to fully elucidate the functional consequences of this regulatory axis. (5) The tumor-suppressive role of *STEAP3-AS1* was not validated in vivo using xenograft models. Animal studies are warranted to clarify its physiological relevance and translational potential. (6) Since antisense lncRNAs can also negatively regulate their sense-strand mRNAs, it remains unclear whether *STEAP3-AS1*’s positive regulation of STEAP3 mRNA extends beyond MDA-MB-231 and MCF-7 cells. Future studies should include a broader range of cell lines, including SK-BR-3, to enhance the generalizability of the conclusions. (7) The precise mechanism by which *STEAP3-AS1* regulates STEAP3 in BC remains to be further validated in conjunction with existing models, including the provision of direct molecular interaction evidence. (8) Although STEAP3 was predicted to be associated with cuproptosis, direct evidence supporting its precise role in this pathway and its relevance to BC biology remains absent. We aim to address this critical gap in greater depth in our future investigations. In summary, this study identifies *STEAP3-AS1* as a tumor-suppressive antisense lncRNA downregulated in BC, where it positively modulates STEAP3 mRNA to inhibit proliferation, migration, and invasion. These findings provide novel insights into BC progression and highlight *STEAP3-AS1*/STEAP3 as potential therapeutic targets and diagnostic biomarkers.

### Conclusions

1. *STEAP3-AS1* is significantly downregulated in BC tissues, and its expression level is associated with the luminal and TNBC subtypes.
2. *STEAP3-AS1* suppresses the proliferative, migratory, and invasive capacities of BC cells.
3. STEAP3 inhibits the migratory and invasive abilities of BC cells.
4. *STEAP3-AS1* exerts a positive synergistic effect on STEAP3 mRNA and may suppress BC cell migration and invasion through STEAP3-mediated mechanisms.

## Abbreviations

BC: breast cancer
BLBC: basal-like breast cancer
BP: biological processes
CCLE: Cancer Cell Line Encyclopedia
DMEM: Dulbecco’s Modified Eagle Medium
ER: estrogen receptor
FBS: fetal bovine serum
GO: Gene Ontology
Her2: human epidermal growth factor receptor-2
HIF: hypoxia-inducible factor
HMMR: hyaluronan-mediated motility receptor
HR: hazard ratio
KEGG: Kyoto Encyclopedia of Genes and Genomes
K-M: Kaplan-Meier
lncRNA: long non-coding RNA
m6A: N6-methyladenosine
MF: molecular functions
MFS: metastasis-free survival
OS: overall survival
P/S: penicillin-streptomycin
PR: progesterone receptor
qRT-PCR: quantitative reverse transcription polymerase chain reaction
si-NC: negative control small interfering RNA
siRNA: small interfering RNA
STRING: Search Tool for Retrieval of Interacting Genes/Proteins
TANRIC: The Atlas of Non-coding RNA in Cancer
TCGA: The Cancer Genome Atlas
TNBC: triple-negative breast cancer
WB: Western blotting

## Declarations

### Availability of data and materials

The datasets analyzed for this study can be found in the TANRIC [https://www.tanric.org], lnCAR [https://lncar.renlab.org/], KEGG [http://www.kegg.jp/], GO [https://geneontology.org/] and STRING [https://cn.string-db.org/]. Other datasets generated for this study are available from the corresponding author on reasonable request.

### Copyright

© The Author(s) 2026.
